# Geographical Pattern and Environmental Correlates of Regional-Scale General Flowering in Peninsular Malaysia

**DOI:** 10.1371/journal.pone.0079095

**Published:** 2013-11-15

**Authors:** Shinya Numata, Masatoshi Yasuda, Ryo O. Suzuki, Tetsuro Hosaka, Nur Supardi Md. Noor, Christine D. Fletcher, Mazlan Hashim

**Affiliations:** 1 Department of Tourism Science, Tokyo Metropolitan University, Hachiouji, Tokyo, Japan; 2 Kyushu Research Center, Forestry and Forest Products Research Institute, Kumamoto, Kumamoto, Japan; 3 Sugadaira Montane Research Center, University of Tsukuba, Ueda, Nagano, Japan; 4 Forestry Division, Forest Research Institute Malaysia, Kepong, Selangor, Malaysia; 5 Institute of Geospatial Science and Technology, Universiti Teknologi Malaysia, UTM, Skudai, Johor Bahru, Malaysia; University of Massachusetts, United States of America

## Abstract

In South-East Asian dipterocarp forests, many trees synchronize their reproduction at the community level, but irregularly, in a phenomenon known as general flowering (GF). Several proximate cues have been proposed as triggers for the synchronization of Southeast Asian GF, but the debate continues, as many studies have not considered geographical variation in climate and flora. We hypothesized that the spatial pattern of GF forests is explained by previously proposed climatic cues if there are common cues for GF among regions. During the study, GF episodes occurred every year, but the spatial occurrence varied considerably from just a few forests to the whole of Peninsular Malaysia. In 2001, 2002 and 2005, minor and major GF occurred widely throughout Peninsular Malaysia (GF2001, GF2002, and GF2005), and the geographical patterns of GF varied between the episodes. In the three regional-scale GF episodes, most major events occurred in regions where prolonged drought (PD) had been recorded prior, and significant associations between GF scores and PD were found in GF2001 and GF2002. However, the frequency of PD was higher than that of GF throughout the peninsula. In contrast, low temperature (LT) was observed during the study period only before GF2002 and GF2005, but there was no clear spatial relationship between GF and LT in the regional-scale episodes. There was also no evidence that last GF condition influenced the magnitude of GF. Thus, our results suggest that PD would be essential to trigger regional-scale GF in the peninsula, but also that PD does not fully explain the spatial and temporal patterns of GF. The coarse relationships between GF and the proposed climatic cues may be due to the geographical variation in proximate cues for GF, and the climatic and floristic geographical variations should be considered to understand the proximate factors of GF.

## Introduction

General flowering (GF) is a supra-annual community-level synchronization of reproduction of many plant taxa in South-East Asian rain forests. In this region, diverse tree species, including dipterocarps, synchronize their reproduction over 5–7 months at irregular intervals of 1–10 years [1]–[6]. For example, 70% of emergent trees and 40% of canopy trees flowered only during GF periods in a Bornean rain forest [3]. GF results in a massive number of fruits and extensive seedling establishment. Conversely, most dipterocarps and many of the other species flower little or not at all in the years between GF episodes. GF can be seen across different forest types (excluding montane and peat swamp forests) at the landscape level [7], and therefore plays a central role in the regeneration of dipterocarp forests in South-East Asia [1], [8].

Several hypotheses have been proposed to explain the mechanism of synchronization at the community level [2], [4], [5]. Some specific climatic conditions, such as low temperature (LT) and prolonged drought (PD), have been proposed as triggers [9]. LT, defined as drops in daily minimum temperature below 20°C during dry spells [1], has been considered over the past two decades as a plausible trigger [3], [4], [10]. In contrast, the importance of severe droughts has been emphasized by many studies since the early 20th century [8], [11]–[13]. In Borneo, irregular prolonged droughts (defined as 30-day running total rainfall of <40 mm) were observed before all GF episodes, although some GFs occurred without a preceding LT [8], [13]. However, the cues and mechanisms of GF remain controversial, because the effects of PD may be obscured by differences in site drainage and plant coverage [1].

Furthermore, intervals of reproduction of trees may influence the pattern of GF. The resource-matching hypothesis explains that the intervals between reproductions are controlled by the consumption of internal resources for flowering and fruiting [14]–[16]. In South-East Asia, the accumulation of phosphorus might be important in determining the occurrence and frequency of reproduction of dipterocarp trees [17]. Isagi *et al.* (1997) suggested that intermittent synchronous production of large seed crops could be tied to the resource balance of plants, even without any inter-annual environmental fluctuations [15]. Satake & Iwasa (2002) also hypothesized that synchronized reproduction can occur over a wide range without environmental fluctuations [16]. Therefore, magnitude of a GF may influence that of the following GF if GF consecutively occurs in a forest.

GF is a community-level phenomenon, but its spatial scale and distribution would be highly variable. Flowering years and seasons can differ among forests and species within a region [7], [18]–[20]. GF can synchronize not only within a region, but also among regions [4], [10], while local GF episodes without inter-regional synchronization are also often observed [1], [21]. Therefore, it has been assumed that global climatic phenomena such as El Niño are associated with severe droughts, which stimulate inter-regional GF [22]–[23]. If Asian dipterocarp species share common climatic cues for synchronized reproduction within and among forests, the spatial and temporal patterns of the cues would be consistent with those of GF. Yasuda *et al.* (1999) found a significant spatial relationship between the magnitude of GF and preceding low night-time temperature during dry spells in the Malay Peninsula in 1996 [4]. However, information on the relationship between spatiotemporal patterns of GF and climatic conditions at the large scale is surprisingly limited due to the lack of dataset of phenology at large-scale.

Understanding of environmental correlates to large-scale GF phenomenon is important for predicting fruit supply for large-scale forest restoration and negative impacts of global climate change on tropical rainforest ecosystem [8], [10], [24]. We monitored the density of fruiting trees in forests to evaluate magnitude of GF phenomenon throughout Peninsular Malaysia from 2001 to 2005. In this study, we hypothesized that: (1) geographical pattern and scale of GF would vary among GF episodes, (2) geographical pattern of GF would be explained by spatial occurrences of last GF, PD and/or LT. To evaluate the spatial and temporal variations of GF, geographical patterns of GF were compared among different episodes. As proximate factors affecting spatial and temporal pattern of GF, we examined the effects of last GF magnitude and two plausible meteorological factors (PD and LT) on the magnitude of GF. Finally, we also examined the characteristics of the critical climatic conditions to discern geographical variations in cues and to predict the effects of global climate change.

## Materials and Methods

### Geographical Distribution of General Flowering in Peninsular Malaysia

Using the methods of Yasuda *et al*. (1999) and Numata *et al*. (2003), we monitored forests from November 2001 to June 2005 in 10 states (except Perlis state) in Peninsular Malaysia [4], [10]. To determine the geographical distribution pattern of GF, we surveyed the density of fruiting dipterocarp trees in forest reserves, protected forests and old secondary forests, from lowlands to hills accessible by vehicles. We established 52–84 observation points for the survey. The latitude, longitude and elevation of each point was recorded on a GPS receiver. The location of the observation point did not necessarily correspond with that of a forest, because observations were made from up to 1 km away with binoculars. No specific permissions were required for our observations because most of the observations were made at forest parks opened to the public or the rest were made from a distance (on the fringe of a forest).

The observation time is important in quantifying the magnitude of GF [4]. Numata et al. (2003) suggested two potential GF seasons (spring and autumn types) characterized by the annual patterns of rainfall and low temperature in Peninsular Malaysia when GF is likely to occur [10]. They assumed that many flowerings occur in both the second (spring type) and fourth (autumn type) quarters in Peninsular Malaysia. Dipterocarp species have a short-flowering period (2–3 week) and do not flower simultaneously with one another in a local species assemblage, but disperse their mature fruits synchronously [25]. Therefore, if spring or autumn type GF occurs, we anticipate that conspicuous red or light yellowish immature fruits can be observed in forests. Consequently, we made observations in June–July (the spring type) and November–December (the autumn type) of 2001–2005. The use of two or more observers minimized observation errors. The GF status of each forest was evaluated by eye and scored on the basis of the density of fruiting dipterocarps as “none”, “fruiting” (a few dipterocarps bearing fruits), “minor” (up to 50%) and “major” (50% or more of large dipterocarps bearing fruits).

### Meteorological Data

The daily minimum temperatures (°C) and daily rainfall (mm) of 14 principal meteorological stations in Peninsular Malaysia from 1981 to 2008 were provided by the Malaysian Meteorological Department. Previous studies have demonstrated that beginning of flowering in GF occurs approximately 2 months after abnormal meteorological condition [1]–[4]. Numata *et al.* (2003) also implied that the dry and cool seasons in Peninsular Malaysia may correspond to trigger period of GF [10]. Based on the rainfall seasonality in Peninsular Malaysia, we focused on relatively dry seasons (3 months) before the beginning of GF episode as a putative trigger period: January–March in 2002–2005 for the spring type GF and June–August in 2001–2004 for the autumn type GF. During the putative trigger periods, the occurrences of abnormal weather conditions, PD (prolonged drought: 30-day moving total rainfall <40 mm [3]) and LT (low temperature: daily minimum temperature <20°C [1]) were determined from the meteorological data. We did not calculate the 30-day moving total of rainfall when rainfall did not occur in the preceding 29 days. To quantify intensity of climatic cues, we calculated numbers of PD days and LT days for each putative trigger period. We also determined monthly occurrences of LT and PD during the putative periods of floral induction from 2001 to 2005. To determine the ENSO (El Niño Southern Oscillation) phase, we used the Niño 3 index and ENSO information from 2001 to 2005 provided by the Japan Meteorological Agency.

### Data Analysis

All statistical analyses were conducted in R v. 2.9.2 statistical software (R Foundation for Statistical Computing, 2010). To examine geographical pattern of GF and factors affecting GF magnitude and distribution, we used a generalized linear mixed model (GLMM) with binomial errors for each GF episode using glmmML package in R. As data for the models, we used numerical transformed GF scores (none: 0; sporadic: 1; minor: 3; major: 5) for each episode. To specify GF status in a proportional response variable, we bind two vectors into a single object: (“GF scores” and “5 - GF scores”). We used forest id as a random effect in the models. The fixed effects of geography (latitude, longitude and elevation) on GF status were examined to determine the geographical pattern of GF. To examine effects of proximate factors on GF status, GLMM was used to test the fixed effects of last GF status and preceding occurrences of PD and LT. For the analyses, forests within 50 km of meteorological stations were chosen. The level of significance was assessed by the Wald test. To analyze correlations between climatic characteristics and frequencies of LT and PD among 14 meteorological stations, we used Spearman’s rank correlation test. To compare frequencies of LT and PD, Fisher’s exact test and Ryan’s multiple comparison method (MCM) were used.

## Results

### Spatial and Temporal Patterns of General Flowering in Peninsular Malaysia

**Figure 1:**
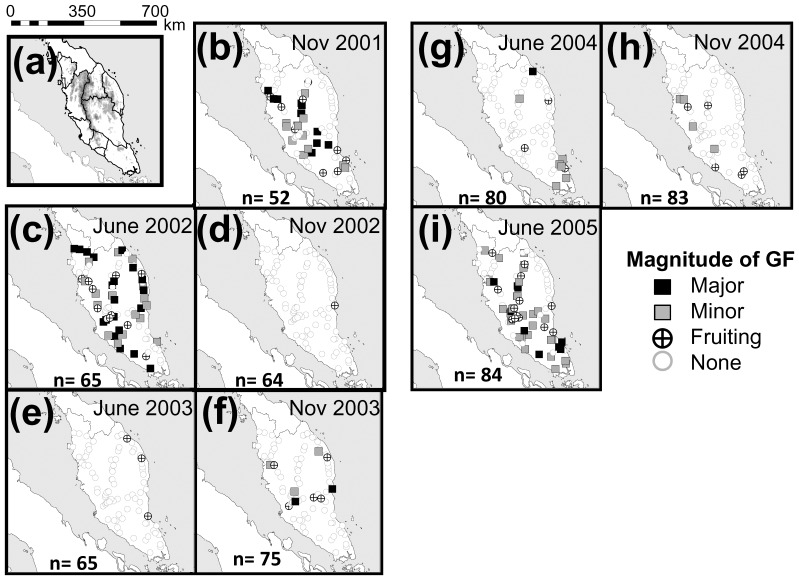
Geographical distribution of GF from November 2001 to June 2005. Each site was scored for the density of fruiting dipterocarp trees (major GF, minor GF, sporadic fruiting or none). (a) Topography of Peninsular Malaysia. (b, d, f, h) GF scores in November–December 2001–2005 (the autumn type). (c, e, g, i) GF scores in June-July 2002–2005 (the spring type).

Different spatial scales of GF were observed during the study period (Figure 1). In 2001, 2002 and 2005, minor and major GF occurred widely throughout Peninsular Malaysia (Figure 1): 44% of forests in November 2001 (GF2001), 59% in June 2002 (GF2002) and 46% in June 2005 (GF2005). Local-scale GF also occurred in November 2003 (7%), June 2004 (9%) and November–December 2004 (5%). We discerned three spatial scales of GF episodes: regional-scale GFs across six or more states (November 2001, June 2002 and June 2005), local-scale GFs with sparse GF in a few states (November 2003, June 2004 and November 2004) and sporadic fruiting episodes (November 2002 and June 2003).

In GF2001, major and minor GF occurred in central and south-eastern Peninsular Malaysia, and the result of GLMM analysis suggested significant latitudinal effect on GF status (Table 1). In GF2005, major and minor GF occurred throughout the region except along the north-eastern coast, and significant latitudinal and elevational effects suggested a distribution at higher elevations. In GF2002, in contrast, major and minor GF occurred throughout Peninsular Malaysia except along the central west coast, but no significant geographical effects were found.

**Table 1:** Effects of geography (longitude, latitude and elevation) on GF status in regional-scale GF episodes examined by generalized linear mixed models with binomial error structure.

	GF2001	GF2002	GF2005
(Intercept)	1.35e+02	−1.82e+01	2.80e+01
Elevation	−6.42e-04	9.43e-04	4.61e-03*
Latitude	−1.40e+00*	4.54e-01	1.02e-00***
Longitude	−1.28e+00	1.61e-01	−4.94e-01

* p<0.05,

*** p<0.001,

The forest id was used as a random effect in the models.

### Temporal Pattern of Prolonged Drought and Low Temperature

Although temperatures on the Malay Peninsula are generally uniform throughout the year, there were differences in meteorological variables among the meteorological stations (Table S1 in File S1). Annual rainfall in 1981–2008 ranged from 1810 mm (Sitiawan) to 2969 mm (Kuantan). Rainfall seasonality (CV of monthly rainfall) also varied from 0.49 (Ipoh) to 1.20 (Kota Bahru). Mean daily minimum temperature in 2001–2005 ranged from 22.9°C (Senai and Kuala Krai) to 25.1°C (Langkawi). Mean 30-day moving total rainfall in 2001–2005. 30-day moving total rainfall ranged from 145.2 mm (Temerloh) to 264.3 mm (Kuantan).

**Figure 2:**
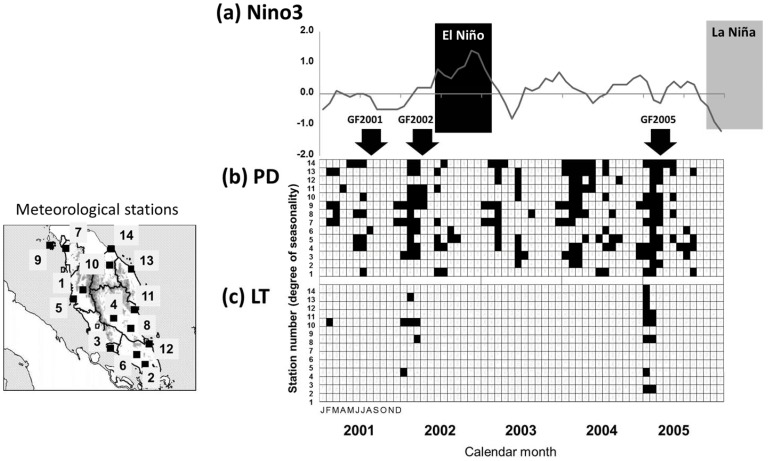
Occurrences of ENSO, PD and LT in Peninsular Malaysia from 2001 to 2005. (a) Niño 3 values as ENSO index, (b) months with PD (30-day moving total rainfall <40 mm) and (c) months with LT (daily minimum temperature <20°C). An El Niño episode occurred from summer 2002 to winter 2002–03, and La Niña from winter 2005 to spring 2006. Arrows indicate regional-scale GF episodes (GF2001, GF2002 and GF2005). Station names are identified in Table 2.

Although frequencies of PD and LT varied greatly among the meteorological stations, PD was much more frequent than LT during the study. PD occurred every year during the study period while LT was not observed during the study period except before GF2002 and GF2005 (Figure 2). In 2001–2005, 2137 days with PD and 43 days with LT were observed at the 14 meteorological stations. 85.8% of PD occurred in the putative trigger period (July–August and January–March). All of LT occurred in the putative trigger period in 2001–2005. Among the 14 stations, frequencies of LT and PD during 2001–2005 were significantly positively correlated with those during 1981–2008 (LT: r_s_ = 0.72, *P*<0.01; PD: r_s_ = 0.93, *P*<0.0001; Table 2), suggesting consistency of our results with long-term regional trends. The long-term averages (±SD) of the frequencies of LT and PD at the 14 stations were 0.0035±0.0053 day^–1^ and 0.0781±0.0508 day^–1^, respectively. This means that PD was >22 times as frequent as LT. The long-term frequency of LT varied greatly among the stations, from 0.0001 day^–1^ (Ipoh and Kluang) to 0.0205 day^–1^ (Kuala Krai) (Table 2). The long-term frequency of PD also varied greatly, from 0.0181 day^–1^ (Ipoh) to 0.1784 day^–1^ (Langkawi).

**Table 2:** Summary of climate at 14 meteorological stations in Peninsular Malaysia.

						Frequency of LT(day^−1^)		Frequency of PD(day^−1^)		
Meteorological station	Long. (E)	Lat. (N)	Alt (m)	Ann. rainfall (mm)(1981–2008)	Rainfall seasonality(CV of mo. rainfall)	2001–2005	1981–2008	2001–2005	1981–2008	Rank correlation between mo lowest 30dMTR and min temperatures (r_s_)
1. Ipoh	101° 06′	4° 34′	40.1	2583	0.49	0	0.0001	0.0296	0.0181	0.068
2. Senai	103° 40′	1° 38′	37.8	2501	0.52	0.0022	0.0047	0.0197	0.0207	0.248***
3. Malacca	102° 15′	2° 16′	8.5	2024	0.54	0	0.0006	0.0947	0.0669	0.02
4. Temerloh	100° 23′	3° 28′	39.1	1949	0.58	0.0011	0.0054	0.0942	0.0682	0.290***
5. Sitiawan	100° 42′	4° 13′	7	1810	0.59	0	0.0008	0.0805	0.078	0.039
6. Kluang	103° 19′	2° 01′	88.1	2196	0.61	0	0.0001	0.0279	0.0336	0.155**
7. Alor Star	100° 24′	6° 12′	3.9	1994	0.65	0	0.0038	0.1218	0.1356	0.514***
8. Muadzam Shaw	103° 05′	3° 03′	33.3	2432	0.71	0.0027	0.0057	0.0723	0.0403	0.176**
9. Langkawi	99° 44′	6° 20′	6.4	2404	0.75	0	0.0004	0.1999	0.1784	0.133*
10. Kuala Krai	102° 12′	5° 32′	68.3	2486	0.85	0.011	0.0205	0.0756	0.0767	0.128*
11. Kuantan	103° 13′	3° 47′	15.3	2969	0.85	0.0033	0.0037	0.0581	0.0565	−0.001
12. Mersing	103° 50′	2° 27′	43.6	2660	0.89	0.0011	0.0003	0.0323	0.0459	−0.038
13. Kulala Terengganu	103° 06′	5° 23′	5.2	2593	1.16	0.0016	0.0018	0.1057	0.1262	0.022
14. Kota Bharu	102° 17′	6° 10′	4.6	2532	1.2	0.0005	0.0015	0.1528	0.1533	0.038

* p<0.05,

** p<0.01,

*** p<0.001.

There were significant differences in the frequencies of PD and LT between the 1980s, 1990s and 2000s, and the frequencies tended to decrease with time. Across the 14 stations, the frequencies of LT were 0.0053 day^–1^ in the 1980s, 0.0037 day^–1^ in the 1990s and 0.0014 day^–1^ in the 2000s, and they differed significantly between decades (*P*<0.0001; test of proportions with Ryan’s MCM; Table S2 in File S1). Frequencies of PD at the 14 stations were 0.0861, 0.0803 and 0.0640 day^–1^, respectively, and they differed significantly between decades (*P*<0.0001; Table S3 in File S1).

During the survey period, an El Niño episode occurred from summer 2002 to winter 2002–03, and La Niña from winter 2005 to spring 2006, and all three regional-scale GF episodes began during the absence of either (Figure 2). There were also significant differences in the frequencies of PD and LT among ENSO conditions (El Niño, La Niña and neutral): The frequency of LT during El Niño (0.0018 day^–1^) was significantly lower than those during La Niña (0.0039 day^–1^) or neutral conditions (0.0036 day^–1^) (*P*<0.0001; test of proportions with Ryan’s MCM; Table S4 in File S1). The frequency of PD during El Niño (0.1084 day^–1^) was significantly higher than that during La Niña (0.0480 day^–1^) and neutral conditions (0.0835 day^–1^) (*P*<0.0001; Table S5 in File S1).

Rainfall seasonality (coefficient of variation of monthly rainfall) at a station was positively correlated with the frequency of PD across all 14 stations (r_s_ = 0.54, *P*<0.05), but not with that of LT (r_s_ = 0.12, *P*>0.05; Figure S1 in File S1). There were no significant correlations between annual rainfall (1981–2008) and frequencies of LT or PD across the 14 stations (vs LT: r_s_ = –0.12, *P*>0.05; vs PD: r_s_ = –0.26, *P*>0.05; Figure S1 in File S1). Seven stations showed significant positive correlations between the monthly lowest 30-day total rainfall and monthly minimum temperature (Table 2).

### Spatial Relationship between General Flowering, Prolonged Drought and Low Temperature

**Figure 3:**
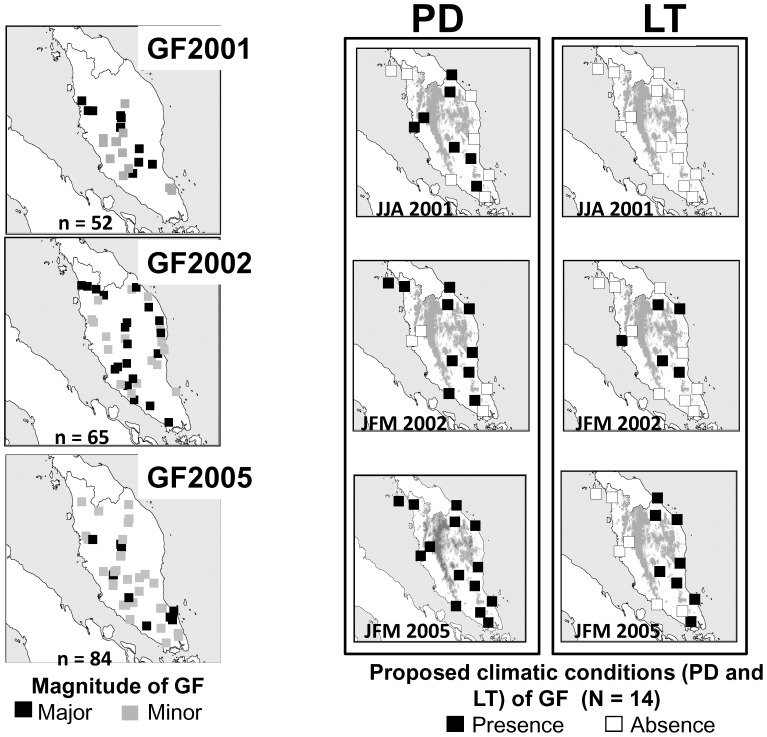
Distribution patterns of major and minor GF (left), and meteorological stations with PD (centre) and LT (right) during the three regional-scale GF episodes. Plots show the presence or absence of PD and LT during the putative floral induction season: June–August (GF2001) or January–March (GF2002 and GF2005).

**Figure 4:**
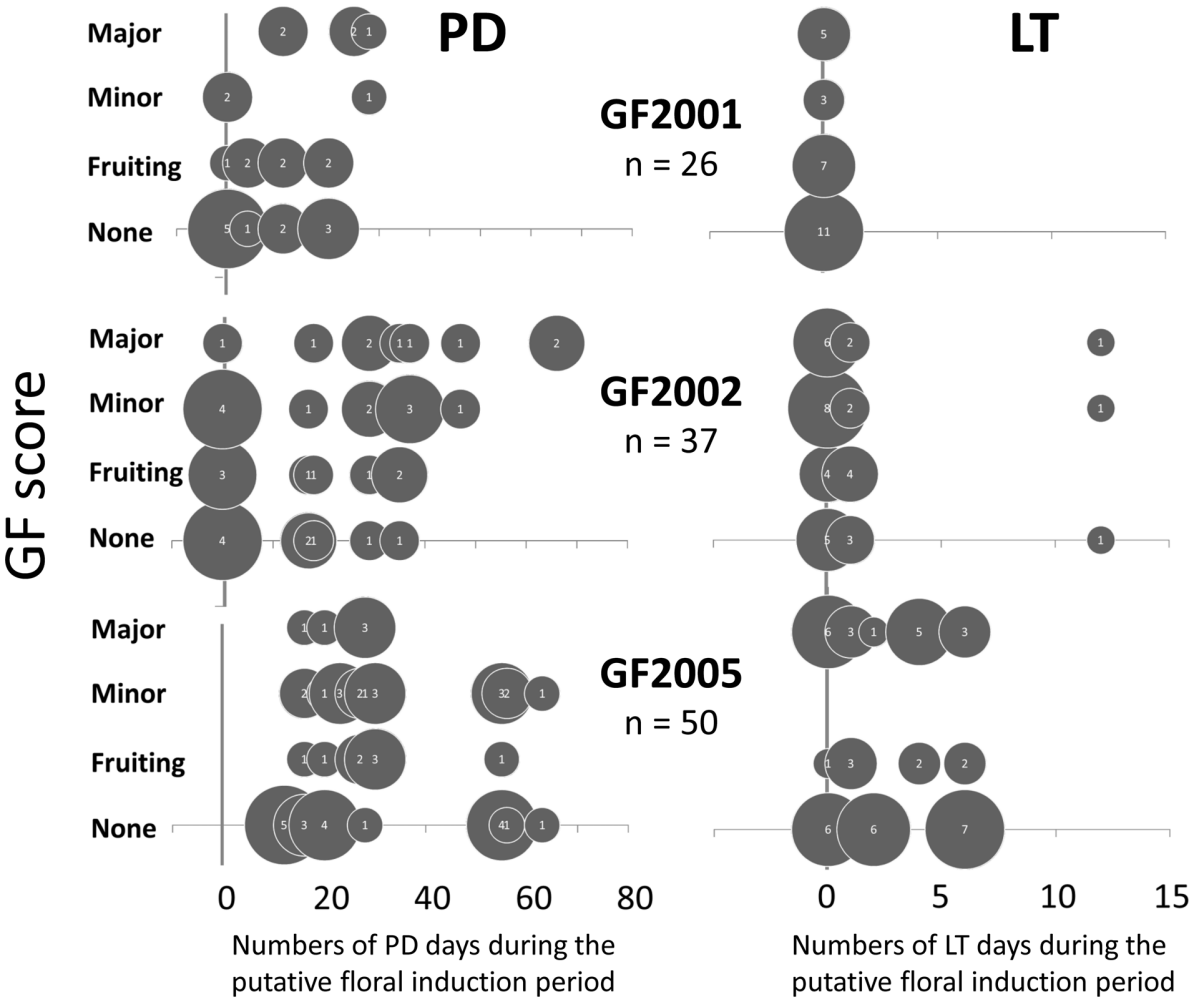
Relationships between GF scores and numbers of PD (left) or LT (right) during the putative trigger periods in GF2001, GF2002 and GF2005. The numbers in the bubbles and bubble’s volumes mean numbers of forests used for the analyses. These analyses used data from meteorological stations within 50 km of sites.

We compared the spatial patterns of GF during the three regional-scale episodes and meteorological stations with PD or LT (Figure 3). In June, July and August 2001, before GF2001, PD was recorded at six stations (Ipoh, Kota Bahru, Kuala Krai, Muadzam Shaw, Sitiawan and Temerloh) in northern and central Peninsular Malaysia, but no LT was recorded. These results suggest that GF tended to occur in areas with PD except in the north in GF2001. In January, February and March 2002, before GF2002, PD was recorded throughout Peninsular Malaysia except at two stations in the central west (Ipoh and Sitiawan) and two in the south (Mersing and Senail), but LT was recorded at only four stations (Kuala Terengganu, Kuala Krai, Temerloh and Muadzam Shaw), in the centre. These results imply that GF tended to occur in areas with PD. In January, February and March 2005, before GF2005, LT was recorded only in the east, but PD was recorded at all stations. In contrast, no clear geographical overlaps between LT and GF were found in any of the three regional-scale episodes. We examined statistically whether GF was associated with last GF status and intensities of preceding occurrences of PD and LT for forests within 50 km of meteorological stations. The results of GLMM analysis suggested that the intensity of PD during the putative trigger period significantly positively influenced GF status in GF2001 and GF2002, but not in GF2005 (Table 3, Figure 4). In contrast, significant effects of last GF status and the intensity of preceding LT on GF scores were not found in any three regional-scale GF episodes (Table 3).

**Table 3:** Effects of last GF status and the two plausible meteorological cues (PD and LT) on GF status.

	GF2001	GF2002	GF2005
(Intercept)	−3.45e+00	−1.31e+00	−1.42e+e00
Last GF scores	–	−3.56e-01	8.41e-01
No. PD days	1.59e-01*	8.40e-02*	1.85e-02
No. LT days	–	−1.22e-01	−1.58e-01

* p<0.05.

Results from GLMM using with binomial error structure were shown. The forest id was used as a random effect in the models.

## Discussion

### Geographical Pattern of General Flowering

GF varied both spatially and temporally. From 2001 to 2005, three regional-scale GF episodes were observed throughout Peninsular Malaysia, although GF was not fully synchronized among forests within the region during these episodes. The regional-scale GF episodes in Peninsular Malaysia would be linked to inter regional synchronization of GF between west and east Malaysia in Malesia. In GF2001, GF2002 and GF2005, GF forests were observed in not only Peninsular Malaysia, but also from Bornean dipterocarp forests [8], [26].

Our results revealed that geographical patterns of GF forests differed between the three regional-scale episodes. The geographical patterns may be partly explained by geographical floristic variation. There are several main forest types with different elevational ranges and edaphic formations where dipterocarp species dominate in the peninsula [27]. The significant positive effect of elevation on GF scores in GF2005 probably resulted from the greater GF scores in hill forest, where hill species such as *Shorea curtisii* dominate, than in lowland forest. In addition, the floristic composition of dominant dipterocarp species in the same forest type may also cause geographical variation, because some dipterocarp species flower more frequently than others [2], ; for example, *Dryobalanops aromatica* flowers annually in Malaysia [21].

### Factors Affecting General Flowering Distribution Pattern

In 2001–2005, GF tended to occur in regions with preceding PD (Figure 3), and the intensity of PD significantly positively influenced the following GF status in GF2001 and GF2002 (Table 3). Therefore, preceding occurrence of PD might be one factor that shapes the spatial pattern of GF. However, the almost annual occurrence of PD throughout Peninsular Malaysia was too frequent to explain the occurrence of GF, suggesting that PD alone does not explain the spatial and temporal patterns of GF. The coarse relationships between GF and the proposed climatic cues might be partly due to the geographical variation in proximate cues for GF. In future studies, we need to examine the definition of PD in terms of climate. For example, previous studies used different definition of PD: Sakai et al. (2006) defined PD as 30-day moving total rainfall <40 mm in a Bornean rainforest (average annual rainfall in 1985–2003: 2722 mm) [8], but Brearly (2007) defined it as in <60 mm in a rainforest of Central Kalimantan (annual rainfall: around 3800 mm) [12].

LT was widely recorded only before GF2002 and GF2005. Therefore, LT is likely associated with regional-scale GF. However, LT was not observed before GF2001 and any other local and sporadic episodes. We also found that LT did not explain the distribution of GF in GF2002 and 2005 (Table 3). Therefore, LT as estimated here does not determine the occurrence and spatial distribution of GF. It has been believed that it is difficult to distinguish between PD and LT because of its correlation [10]. However, the decreasing frequency of LT during the 2000s might have obscured any spatial relationship between PD and LT in the three regional-scale episodes.

Sakai et al. (2006) implied that magnitude of GF may be influenced the level of accumulated resources in addition to the intensity of the trigger [8]. However, no significant effects of last GF status suggest that the resource condition of trees would not be more decisive than climatic cues even if it has an effect. In Japanese beech trees (*Fagus crenata*), for example, it is likely that climatic variation is more critical to controlling the amount of flowering than resource condition, while the quantity of seed production in a year is negatively correlated with that in the preceding year [29].

The coarse relationships between GF and the previously proposed climatic cues may be also due to the poor spatial representativeness of the meteorological stations. Rainfall and temperature are spatially and temporally heterogeneous even in the peninsula [30], [31], and rainfall spatial variability generally increases with distance in the peninsula [32], [33]. Furthermore, differences in observations between flowering and fruiting may influence the association between GF and climatic cues, because factors affecting the density of flowering differ from those affecting the density of fruiting [29]. Percentages of flowering and fruit production are generally highly correlated, even in dipterocarp species [8], [13], but more data on flowering are needed to settle the dispute on climatic cues of GF.

### Trends in Meteorological Cues: Past and Future

There were also considerable geographical variations in the occurrences and frequencies of the proposed climatic cues. The frequencies of PD were significantly correlated with the seasonality of rainfall among the 14 meteorological stations. Peninsular Malaysia lies to the south of the Kangar–Pattani line in the Indo-Sundaic region of South-East Asia, and its vegetation is thought to be aseasonal rain forest [34]. In general, frequent flowering and fruiting in a region with strong rainfall seasonality is well known, because most species, including dipterocarps, flower and fruit annually in seasonal forests in South-East Asia [21], [27]. Comparison of frequencies of PD between seasonal and aseasonal rainforests would be important to understand latitudinal transition from annual flowering and GF as well as its mechanism.

The correlation between PD and LT was discussed in many studies [1], [4], [8], [10], [13], but its geographical variation has not been well considered. In this study, significant positive correlations between monthly lowest minimum temperatures and monthly lowest 30-day running totals of rainfall were found at only seven of the 14 meteorological stations, in central and north-western parts of Peninsular Malaysia. Therefore, contribution of LT to cues for GF might geographically vary even if LT is responsible for GF.

The frequencies of PD and LT decreased from the 1980s to the 2000s. If PD and LT is associated with regional-scale GF, projected increases in temperature (1.1–3.6°C) in Peninsular Malaysia may reduce the frequency of LT events for both seasonal types of GF [35]; in contrast, rainfall variability in Peninsular Malaysia is likely dependent on model choice, and there is no clear trend, owing to the high variability in the precipitation-modulating factor. Therefore, if LT is associated with specific PD-related conditions for GF, increases in temperature would result in decreases in regional-scale GF episodes regionally. Further study is needed to understand how climate change affects frequency and magnitude of PD and LT as well as relationship between LT and PD.

ENSO-related droughts in Borneo may have serious simultaneous impacts on the regeneration of dipterocarp forests by GF episodes under global climate change [8], [22]. In earlier studies of association of GF with ENSO, no simple association was found [1], [4], [5], [20], [22]. However, our results reveal that regional-scale GF in Peninsular Malaysia was triggered during the absence of El Niño and La Niña. This result is consistent with previous studies suggesting that GF was induced at the transition between El Niño and La Niña [8], [10], [36]. Our results also explain why the ENSO-neutral condition important for regional-scale GF episodes: the frequency of PD was higher under El Niño than under neutral and La Niña conditions, but the frequency of LT was lower. Therefore, GF would be favoured under ENSO-neutral conditions in a region where LT is associated with climatic cues for GF. Complex Coupled Global Circulation Models suggest that ENSO frequency and magnitude under global warming are still unclear [37]. In addition, the ENSO–rainfall relationship may vary geographically, even within a region [38], [39]. Further study is required to examine the combined effect of global climate change and ENSO on GF.

### Concluding Remarks for Future Study

Although PD and LT do not fully explain the spatial and temporal patterns of GF in Peninsular Malaysia, PD was likely to trigger GF during the study period. However, it is still unclear why PD can explain only a part of GF occurrences in Peninsular Malaysia. The coarse relationships between GF and the proposed climatic cues may be due to the geographical variation in proximate cues for GF. Thus, information on floristic and phylogeographic regions should also be included to explain the spatial and temporal pattern of GF. Furthermore, intra-specific variation in the response of trees to the environment may also cause different geographical pattern in responses to climate [40]–[42]; for example, geographical variation in masting behaviour occurs in Japanese beech, and its populations in northern Japan can be classified into five [43] or seven regional groups [29]. Furthermore, considering the two different monsoon activities [10], there may be different mechanisms for triggering GF between the spring and autumn types. Both experimental and theoretical approaches focusing on internal and external proximate cues may be required to explore the mechanisms of inter- and intra-regional reproductive synchronization in Southeast Asian dipterocarp forests.

## Supporting Information

File S1 **Combined Supplementary file containing the following items: Table S1, Table S2, Table S3, Table S4, Table S5 and Figure S1.** Table S1 Summary of minimum temperature and 30-day moving total rainfall at the 14 meteorological stations. Table S2 Frequencies of LT in 1980’s, 1990’s, 2000’s and the entire period (1981–2008). Table S3 Frequencies of PD in 1980’s, 1990’s, 2000’s and the entire period (1981–2008). Table S4 Frequencies of LT in neutral, El Niño and La Niña periods. Table S5 Frequencies of PD in neutral, El Niño and La Niña periods. Figure S1. Relationships between frequencies of the proposed climatic cues (LT and PD), annual rainfall, and rainfall seasonality across the 14 meteorological stations.
(DOC)
