# Supplementary material for: Geographical Pattern and Environmental Correlates of Regional-Scale General Flowering in Peninsular Malaysia
Source: PLoS One. 2013 Nov 15;8(11):e79095. doi: 10.1371/journal.pone.0079095 (PMC3829834; doi:10.1371/journal.pone.0079095)
Supplement: File S1 — Combined Supplementary file containing the following items: Table S1, Table S2, Table S3, Table S4, Table S5 and Figure S1. Table S1 Summary of minimum temperature and 30-day moving total rainfall at the 14 meteorological stations. Table S2 Frequencies of LT in 1980’s, 1990’s, 2000’s and the entire period (1981–2008). Table S3 Frequencies of PD in 1980’s, 1990’s, 2000’s and the entire period (1981–2008). Table S4 Frequencies of LT in neutral, El Niño and La Niña periods. Table S5 Frequencies of PD in neutral, El Niño and La Niña periods. Figure S1. Relationships between frequencies of the proposed climatic cues (LT and PD), annual rainfall, and rainfall seasonality across the 14 meteorological stations. (DOC) [file pone.0079095.s001.doc]

Table S1 Summary of minimum temperature and 30-day moving total rainfall at the 14 meteorological stations.

|  |  |  | Daily minimum temperature  (mean + SD) | | | | 30-day moving total rainfall  (mean + SD) | | | |
| --- | --- | --- | --- | --- | --- | --- | --- | --- | --- | --- |
| Meteorological station | Ann. rainfall (mm) (1981-2008) | Rainfall seasonality (CV of mo. rainfall) | 2001-2005 | No. missing values | 1981-2008 |  | 2001-2005 | No. missing values | 1981-2008 |  |
| 1. Ipoh | 2583 | 0.49 | 24.1 + 0.9 | 0 | 23.6 + 1.0 |  | 209.6 + 107.9 | 0 | 211.5 + 103.9 |  |
| 2. Senai | 2501 | 0.52 | 22.9 + 0.8 | 0 | 22.8 + 0.8 |  | 209.2 + 95.8 | 0 | 205.4 + 110.0 |  |
| 3. Malacca | 2024 | 0.54 | 24.1 + 0.8 | 0 | 23.6 + 0.9 |  | 159.5 + 86.2 | 0 | 166.1 + 88.7 |  |
| 4. Temerloh | 1949 | 0.58 | 23.2 + 0.9 | 0 | 23.0 + 0.9 |  | 145.2 + 94.6 | 0 | 160.0 + 95.2 |  |
| 5. Sitiawan | 1810 | 0.59 | 23.6 + 0.8 | 0 | 23.3 + 0.9 |  | 146.1 + 94.0 | 0 | 148.8 + 88.3 |  |
| 6. Kluang | 2196 | 0.61 | 23.4 + 0.7 | 0 | 23.1 + 0.8 |  | 165.3 + 93.1 | 0 | 180.3 + 109.6 |  |
| 7. Alor Star | 1994 | 0.65 | 24.0 + 1.0 | 9* | 23.7 + 1.1 |  | 175.7 + 113.0 | 12** | 163.8 + 107.2 |  |
| 8. Muadzam Shaw | 2432 | 0.71 | 23.1 + 0.9 | 0 | 22.8 + 0.9 |  | 185.4 + 123.8 | 0 | 200.5 + 143.0 |  |
| 9. Langkawi | 2404 | 0.75 | 25.1 + 1.0 | 0 | 24.8 + 1.1 |  | 199.2 + 161.1 | 0 | 200.7 + 151.4 |  |
| 10. Kuala Krai | 2486 | 0.85 | 22.9 + 1.0 | 0 | 22.6 + 1.0 |  | 200.4 + 159.2 | 0 | 204.0 + 171.3 |  |
| 11. Kuantan | 2969 | 0.85 | 23.5 + 1.0 | 0 | 23.2 + 1.0 |  | 264.3 + 247.1 | 0 | 243.8 + 205.3 |  |
| 12. Mersing | 2660 | 0.89 | 23.7 + 1.0 | 0 | 23.4 + 1.1 |  | 211.6 + 163.8 | 0 | 218.2 + 194.6 |  |
| 13. Kulala Terengganu | 2593 | 1.16 | 23.8 + 1.0 | 0 | 23.6 + 1.1 |  | 229.2 + 227.0 | 0 | 212.9 + 252.1 |  |
| 14. Kota Bharu | 2532 | 1.2 | 24.2 + 1.0 | 0 | 23.8 + 1.1 |  | 193.1 + 182.6 | 0 | 207.5 + 253.2 |  |

*19-27 December 2005

**20-31 December 2005

Table S2 Frequencies of LT in 1980’s, 1990’s, 2000’s and the entire period (1981-2008).

| LT | Alor Star | Ipoh | K Terengganu | Kluang | Kota Bharu | Kuala Krai | Kuantan | Langkawi | Malacca | Mersing | Muadzam Shaw | Senai | Sitiawan | Temerloh | All stations |
| --- | --- | --- | --- | --- | --- | --- | --- | --- | --- | --- | --- | --- | --- | --- | --- |
| 1980's | 0.00669 | 0.00030 | 0.00243 | 0.00030 | 0.00183 | 0.03122 | 0.00548 | 0.00000 | 0.00152 | 0.00000 | 0.01066 | 0.00974 | 0.00152 | 0.01156 | 0.0053 |
| 1990's | 0.00466 | 0.00000 | 0.00192 | 0.00000 | 0.00219 | 0.02327 | 0.00383 | 0.00082 | 0.00027 | 0.00027 | 0.00602 | 0.00329 | 0.00082 | 0.00383 | 0.0037 |
| 2000's | 0.00000 | 0.00000 | 0.00091 | 0.00000 | 0.00030 | 0.01156 | 0.00182 | 0.00000 | 0.00000 | 0.00061 | 0.00182 | 0.00122 | 0.00000 | 0.00091 | 0.0014 |
| 1981-2008 | 0.00382 | 0.00010 | 0.00176 | 0.00010 | 0.00147 | 0.02053 | 0.00372 | 0.00038 | 0.00059 | 0.00029 | 0.00571 | 0.00469 | 0.00078 | 0.00538 | 0.0034 |

Table S3 Frequencies of PD in 1980’s, 1990’s, 2000’s and the entire period (1981-2008).

| LT | Alor Star | Ipoh | K Terengganu | Kluang | Kota Bharu | Kuala Krai | Kuantan | Langkawi | Malacca | Mersing | Muadzam Shaw | Senai | Sitiawan | Temerloh | All stations |
| --- | --- | --- | --- | --- | --- | --- | --- | --- | --- | --- | --- | --- | --- | --- | --- |
| 1980's | 0.1582 | 0.0286 | 0.1828 | 0.0316 | 0.1847 | 0.0790 | 0.0578 | 0.1680 | 0.0675 | 0.0520 | 0.0293 | 0.0243 | 0.1086 | 0.0724 | 0.0861 |
| 1990's | 0.1520 | 0.0063 | 0.1013 | 0.0474 | 0.1495 | 0.0895 | 0.0685 | 0.1906 | 0.0728 | 0.0526 | 0.0446 | 0.0244 | 0.0712 | 0.0531 | 0.0803 |
| 2000's | 0.0944 | 0.0207 | 0.0973 | 0.0204 | 0.1262 | 0.0611 | 0.0420 | 0.1676 | 0.0596 | 0.0322 | 0.0432 | 0.0131 | 0.0550 | 0.0639 | 0.0640 |
| 1981-2008 | 0.1356 | 0.0181 | 0.1262 | 0.0336 | 0.1533 | 0.0767 | 0.0565 | 0.1784 | 0.0669 | 0.0459 | 0.0403 | 0.0207 | 0.0780 | 0.0628 | 0.0766 |

Table S4 Frequencies of LT in neutral, El Niño and La Niña periods.

|  | Alor Star | Ipoh | K Terengganu | Kluang | Kota Bharu | Kuala Krai | Kuantan | Langkawi | Malacca | Mersing | Muadzam Shaw | Senai | Sitiawan | Temerloh | All stations |
| --- | --- | --- | --- | --- | --- | --- | --- | --- | --- | --- | --- | --- | --- | --- | --- |
| Neutral | 0.00411 | 0.00000 | 0.00149 | 0.00000 | 0.00149 | 0.02162 | 0.00467 | 0.00070 | 0.00019 | 0.00056 | 0.00568 | 0.00504 | 0.00093 | 0.00560 | 0.003574 |
| La Niña | 0.00484 | 0.00032 | 0.00290 | 0.00000 | 0.00193 | 0.02018 | 0.00322 | 0.00000 | 0.00161 | 0.00000 | 0.00740 | 0.00547 | 0.00097 | 0.00612 | 0.003898 |
| El Niño | 0.00113 | 0.00000 | 0.00057 | 0.00057 | 0.00057 | 0.01726 | 0.00170 | 0.00000 | 0.00000 | 0.00000 | 0.00160 | 0.00227 | 0.00000 | 0.00340 | 0.001798 |

Table S5 Frequencies of PD in neutral, El Niño and La Niña periods.

|  | Alor Star | Ipoh | K Terengganu | Kluang | Kota Bharu | Kuala Krai | Kuantan | Langkawi | Malacca | Mersing | Muadzam Shaw | Senai | Sitiawan | Temerloh | All stations |
| --- | --- | --- | --- | --- | --- | --- | --- | --- | --- | --- | --- | --- | --- | --- | --- |
| Neutral | 0.151476 | 0.011207 | 0.11879 | 0.041838 | 0.175756 | 0.081496 | 0.053418 | 0.193195 | 0.078446 | 0.051737 | 0.047059 | 0.029511 | 0.086104 | 0.067239 | 0.083474 |
| La Niña | 0.095052 | 0.015127 | 0.086901 | 0.023817 | 0.068233 | 0.064594 | 0.025748 | 0.150566 | 0.037657 | 0.020921 | 0.023817 | 0.008046 | 0.035404 | 0.032507 | 0.047974 |
| El Niño | 0.157984 | 0.044168 | 0.218007 | 0.026048 | 0.234994 | 0.087099 | 0.120045 | 0.189616 | 0.083239 | 0.071914 | 0.054918 | 0.016421 | 0.128539 | 0.102492 | 0.108444 |


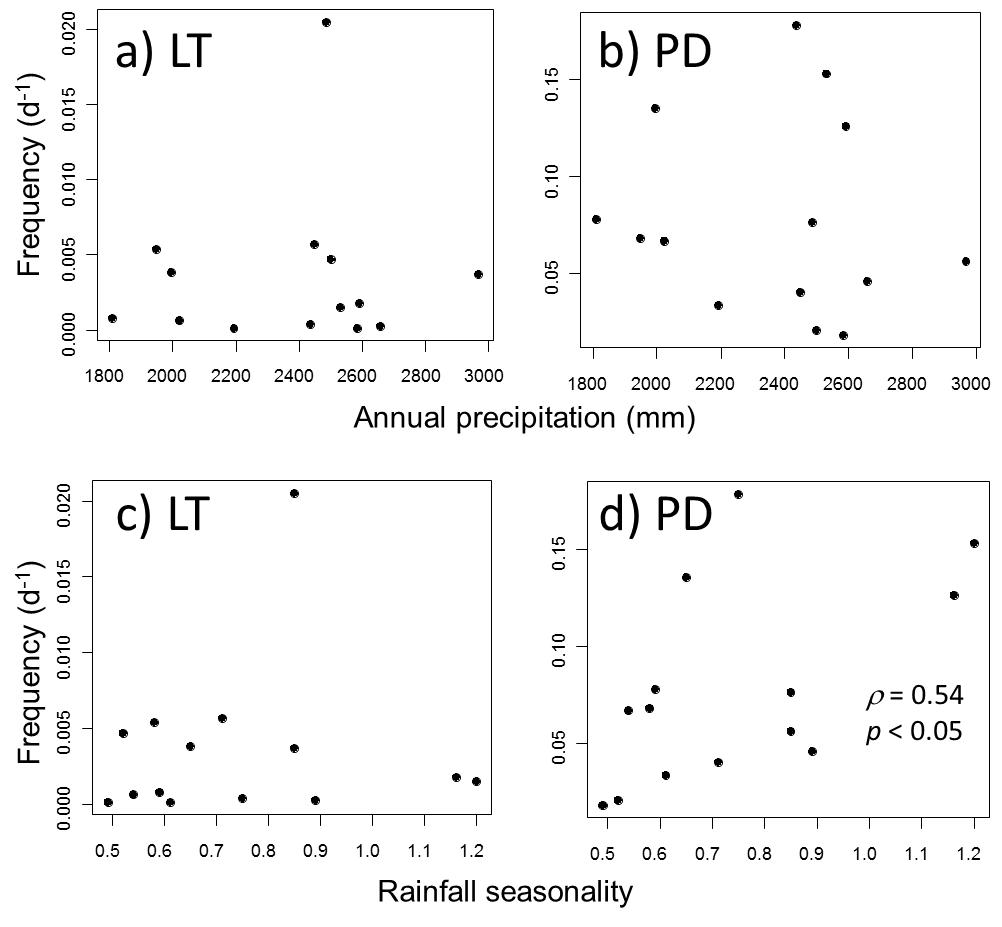


Figure S1. Relationships between frequencies of the proposed climatic cues (LT and PD), annual rainfall, and rainfall seasonality across the 14 meteorological stations.(a) annual rainfall vs. LT, (b) annual rainfall vs. PD, (c) rainfall seasonality vs. LT, and (d) rainfall seasonality vs. PD.
